# Characterization of garlic oil/β-cyclodextrin inclusion complexes and application

**DOI:** 10.3389/fnut.2023.1308787

**Published:** 2023-11-29

**Authors:** Shangjian Li, Jiajia Chen, Yuntong Liu, Honghao Qiu, Wei Gao, Kundian Che, Baogang Zhou, Ran Liu, Wenzhong Hu

**Affiliations:** ^1^School of Pharmacy and Food Science, Zhuhai College of Science and Technology, Zhuhai, China; ^2^College of Life Science, Jilin University, Changchun, China; ^3^Zhuhai Livzon Microsphere Technology Co. Ltd., Zhuhai, China; ^4^College of Life Science, Dalian Minzu University, Dalian, China

**Keywords:** garlic oil, controlled release, characterization, inclusion complexes, β-cyclodextrin

## Abstract

Garlic oil is a liquid extracted from garlic that has various natural antibacterial and anti-inflammatory properties and is believed to be used to prevent and treat many diseases. However, the main functional components of garlic oil are unstable. Therefore, in this study, encapsulating garlic oil with cyclodextrin using the saturated co-precipitation method can effectively improve its chemical stability and water solubility and reduce its characteristic odor and taste. After preparation, the microcapsules of garlic oil cyclodextrin were characterized, which proved that the encapsulation was successful. Finally, the results showed that the encapsulated garlic oil still had antioxidant ability and slow-release properties. The final addition to plant-based meat gives them a delicious flavor and adds texture and mouthfeel. Provided a new reference for the flavor application of garlic cyclodextrin micro-capsules in plant-based meat patties.

## 1 Introduction

Garlic (*Allium sativum* L.) is an aromatic annual herbaceous spice and one of the ancient, essential plants widely used as food, spice, and medicine since ancient times (1, 2). Garlic and its active compounds are believed to lower the risk of diabetes and cardiovascular disease by strengthening the immune system to prevent infection (3). Garlic is frequently used to enhance cardiovascular health, bolster the immune system, and prevent cancer. Garlic’s potential antibacterial, anti-inflammatory, and antioxidant effects can reduce the risk of cardiovascular ailments. Conversely, the anti-tumor effects of garlic may help prevent various types of cancer (3, 4). Garlic is also antibacterial, anti-fungal, anti-aging and anti-cancer (5). Garlic oil (GO) is a naturally derived vegetable oil from garlic. It has commonly been associated with numerous health benefits (6). GO mainly consists of sulfides such as diallyl sulfide (DAS), diallyl disulfide (DADS), diallyl trisulfide (DATS), diallyl tetrasulfide (DATTS) and other similar compounds (7, 8). Garlic oil (GO) preserves several of garlic’s physiological functions and has good antioxidant qualities (9). DADS, DATS and DAS have all been reported to have demonstrated cancer-inhibiting properties (10–12). Additionally, GO finds extensive usage in the food sector (13). I In food, it enhances flavor, aroma, and has preservative effects (14). Despite its efficacy, some negatives restrict the wide-scale use of GO in the food industry (15). The active components of GO are vulnerable to reduced activity resulting from light, oxygen, heat, and moisture, which reduces their nutritional and pharmacological value (16, 17). GO exhibits low solubility in water (18). Although garlic oil has many benefits, too high concentrations produce strong and pungent odors and tastes that affect people’s interest in food and its sensory quality (19). It is becoming very popular to study how to encapsulate garlic oil so that it retains its activity while reducing its flavor (18, 20, 21).

Beta-cyclodextrin (β-CD) is a natural cyclodextrin that has numerous applications (22). β-CD is a colorless, odorless, and crystalline chemical compound containing seven glucose molecules bound together in a ring structure (23, 24). Beta-cyclodextrin is a hydrophilic compound that can create internal inclusions in solution, also called “molecular containers,” because of its molecular structure (25). The incorporation of β-CD has several functions, including enhancing drug bioavailability, modulating drug release rates, improving the stability of dissolved substances in water, and enhancing the efficiency of catalytic reactions (26). β-CD finds application in diverse industries such as food, cosmetics, pharmaceuticals and materials (27). β-CD can enhance the stability and bioavailability of vitamins, flavonoids, and phenolic acids (28, 29). The use of β-CD to encapsulate volatile substances with unstable physicochemical properties, like essential oils, has been widely researched in recent years (30). Moreover, β-CD efficiently enhances the delivery properties of processed composite ingredients in food systems. It also modifies the texture and density of food products, thus ameliorating their mouthfeel.

For this reason, we selected β-CD as an encapsulating agent for GO to enhance its stability, conceal unpleasant odors, and increase its water solubility, thereby improving its utility. GO, and β-CD inclusion complexes were formed through co-precipitation. The samples are characterized by different methodological, analytical techniques such as Ultraviolet visible spectroscopy (UV–Vis), Scanning Electron Microscope (SEM), Fourier Transform Infrared (FT-IR), Thermogravimetric Analysis (TG), differential scanning calorimetry (DSC), and X-ray diffraction (XRD). The retention and release characteristics of the inclusion compounds were measured to verify their antioxidant capacity. Finally, added to the preparation of plant-based meat patties, the detector evaluates the textural and organoleptic structure of the patties.

## 2 Materials and methods

### 2.1 Materials

β-Cyclodextrin (99%, CAS: 7585-39-9, Shanghai Yuanye Biotechnology Co. Ltd); Garlic oil (90%, Ji’an Huashuo Spice Oil Co.); Plant-based meat patties are provided by the Pharmacy and Food Science Laboratory of Zhuhai University of Science and Technology. Ingredients (soy protein, pea protein, gluten meal, carrageenan, inulin, starch acetate).

### 2.2 Instruments and equipment

Scanning Electron Microscope (TESCAN MIRA, TESCAN, Czech Republic); Ultrasound-microwave assisted extraction (UMAE) device (XH-300A, Xianghu, China); UV Spectrophotometer (Shimadzu, UV-2600i, Japan); Frozen centrifuges (1248R, Genecompany, HK); Differential Scanning Calorimeter (DSC 214 Polyma, netzsch, Germany); Fourier Transform infrared spectroscopy (Shimadzu, IRAffinity-1S, Japan); X-ray Diffractometers (Bruker, D8 DISCOVER, Germany); Texture analyzer (Bosin, TA. TOUCH, China); GC–MS (Shimadzu, QP-2010, Japan).

### 2.3 Preparation of CD-GO

Weigh 10% (w/w) β-cyclodextrin and add 100 grams of ultrapure water. Stir the mixture at 80°C until it is clear. Disperse the GO with anhydrous ethanol, and slowly add it dropwise to the cyclodextrin solution. The mixture was dispersed in an ultrasonic device at 550 W for 1 h and then sealed with plastic wrap. Finally, place the mixture in a refrigerator at 4°C. After 12 h, the mixture underwent filtration and was subsequently dried at a temperature of 60°C for a duration of 24 h, resulting in the production of CD-GO microencapsulated powder.

### 2.4 GC–MS analysis

Sample preparation: Trace amounts of 1 g of raw β-CD, GO and CD-GO were dissolved separately and diluted with 100 mL of anhydrous ethanol. Cyclodextrin microcapsules were disrupted by ultrasound. The supernatant was removed by centrifugation, filtered through a 0.22 μ microporous membrane and loaded into 1 mL sample vials for complete GC–MS scanning. Raw β-CD, GO and CD-GO were analyzed by GC-ΜS using GCMS-QP2010 (31).

The GC section was set up as follows: Rtx-5MS column (30 m × 0.28 mm × 0.25 μm); carrier gas was He (99.999% purity); inlet temperature 240°C; split injection with AOC-20i, viscosity compensation 0.3 s, injection volume 1 μL, split ratio 50; flow rate controlled by pressure, pressure 22 Kpa, total flow rate 59.6 mL/min, column flow rate 1.11 mL/mi, starting column temperature 30°C, The temperature is ramped up to 200°C at a rate of 10°C/min and held for 3 min. The MS section was set up as follows: electron bombardment ion source, energy 70 eV; detector gain 0.2 kV; ion source 230°C; interface temperature 250°C; full scan mode, mass scan range 20–800 amu; solvent delay 1.5 min.

### 2.5 Physical properties

To determine the bulk density, take an appropriate amount of CD-GO powder, weigh it accurately and place it in a graduated 100 mL measuring cylinder, shake the powder up and down, read the volume of the powder in the cylinder and calculate the bulk density by weight according to the density formula (32). The resting angle method was used to determine the fluidity of CD-GO. A funnel was placed on an iron stand, then a certain height (H) of microencapsulated powder was added to the funnel, and the powder was injected into the center of a disk of finite diameter (R) until the material on the beveled edge of the powder accumulation layer flowed out automatically along the edge of the disk, and the injection was stopped, and the angle of repose was calculated according to the following formula θ. For the determination of the moisture content, the water content of the inclusions was determined by drying a 1 g sample of powder in a glass weighing bottle at 105°C until a constant weight was obtained. The moisture content of the powder was calculated from the weight loss (%).

For the hygroscopicity test, a 1 g sample of the inclusion (three replicates) was placed in a petri dish in a sealed container containing a saturated solution of Na2SO4 at 25°C and 81% relative humidity (RH). The samples were weighed after 1 week, and hygroscopicity was defined as the number of grams of water absorbed per 100 g of powder (32).

### 2.6 GO standard curve

Four vials of standard solutions at known concentrations were prepared by diluting GO with anhydrous ethanol and analyzed by GC–MS under the conditions set out in 2.2. The results of 2.2 were used to set up a table of components, and the MS conditions were changed from SCAN mode to SIM mode for quantitative determination. The peak height was used as an indicator to make a GC–MS standard curve for GO.

### 2.7 Encapsulation rate

A suspension was prepared by mixing 1 g of CD-GO with 10 mL of anhydrous ethanol. The supernatant was removed by centrifugation at 100 W per second at 1 s intervals and filtered through a 0.22 μm microporous membrane.


$$EE\%=\frac{M1}{M2}\times 100\%$$

Where: M2 is the total amount of GO added; M1 is the X content of CD-GO.

### 2.8 Particle size distribution

The particle size analysis of the Raw β-CD, recrystallized β-CD, CD-GO, and physical mixtures was measured using the solids module of the laser particle size analyzer, set at room temperature (25°C), with a scattering angle of 90°C; the optical model selected: Fraunhofer.rf780f, three measurements, 100 runs per measurement The particle size and particle number and the size and volume distribution were recorded in 3 runs.

### 2.9 Fourier transform infrared spectrometer

The infrared spectra of the samples to be measured (Raw β-CD, GO, physical mixtures, and CD-GO) were scanned with an FTIR in the mid-infrared region in the wave number range 4,000 to 400 cm^−1^ and with a spectral resolution of 4 cm^−1^. Before the measurements, the solid samples (Raw β-CD, physical mixture, and CD-GO) were ground and mixed thoroughly with KBr. KBr sheets were then made by passing them through a hydraulic press. Samples are prepared using the KBr windowing technique. Wait until the test is complete and save the spectrum for the subsequent analysis.

### 2.10 Scanning electron microscope

Following removing the samples from the vacuum drying oven, a conductive coating was applied to their surface. Subsequently, the surface morphology was assessed using a scanning electron microscope.

### 2.11 Thermal analysis

Thermogravimetric analysis (TG) was carried out in a DTG-60H analyzer using ap-proximately 3–5 mg of sample in an aluminum crucible under a dynamic nitrogen atmosphere at a heating rate of 5°C/min over a temperature range of room temperature to 400°C. After the test, the data was saved for subsequent analysis.

Differential scanning calorimetric (DSC) was carried out in a DSC214 analyzer by weighing approximately 10 mg of samples with a physical mixture in an aluminum crucible under a dynamic nitrogen atmosphere, with the empty crucible serving as a control and the parameters calibrated manually. The scanning temperature range was 0°C to 100°C, and the rate was 10 C/min. The starting temperature (To), final temperature (Tc) and peak temperature (Tp) of the analysis were recorded by STARe thermal analysis software.

### 2.12 X-ray diffraction

An appropriate amount of sample was weighed, ground appropriately and scanned using X-ray diffraction in the 2θ range of 5–30°, setting the scan rate at 10°/min and saving the data for subsequent analysis.

### 2.13 Retention rate

Storage at 37°C and the GO content of CD-GO was measured daily for 30 consecutive days. Retention of pure CA was calculated by recording daily the mass loss over 30 days. The retention rate was calculated using the formula.


$$RR\left(\%\right)=\frac{C_{n}}{C}$$

The formula $C_{n}$ is the concentration on day n, and C is the initial concentration. The pure GO formula then becomes the weight on day n divided by the initial weight.

### 2.14 Release performance

0.2 g of the CD-GO complex was weighed and suspended in 14 mL of phosphate-buffered solution (PBS, 0.01 M, pH = 7.4) in a centrifuge tube, which was then incubated in a water bath at a set temperature while shaking at about 200 rpm for 0–12 h. Samples are then taken every 2 h for GO concentrations.

### 2.15 Texture profile analysis

0, 2, 4, 6, 8, and 10% CD-GO was added to the plant-based meat preparation process, and the resulting product was tested for Texture profile analysis using a tetrameter.

### 2.16 Meatloaf structure

The samples were dehydrated and dried for pre-treatment and then observed under SEM.

### 2.17 Sensory evaluation

This study was reviewed and approved by the Research Ethics Committees of the Zhuhai College of Science and Technology. All participants signed written informed consents. Document number for human ethics: (2023) Session Audit No. (2023–016). Volunteers were selected from students at Zhuhai College of Science and Technology. Informed consent was obtained prior to study initiation. A nine-point sensory evaluation was conducted on a blank group with no added β-cyclodextrin, a control group with added physical mixture, and an experimental group with added CD-GO. The meat patties were deep-fried, and 10 individuals were chosen as experimental evaluators. The diverse plant meat formulations were evaluated using a nine-point preference scale based on their appearance, taste, odor, degree of organization, and chewiness. The rating scale consisted of9 was extremely like; 8 was very like; 7 was very like; 6 was a little like; 5 was talk about like or dislike; 4 was slightly dislike; 3 was dislike; 2 was very dislike; and 1 was not like at all.

## 3 Results

### 3.1 Characterization of garlic oil/β-cyclodextrin inclusion complexes

#### 3.1.1 Preparation of CD-GO and its physical properties

**Figure 1:**
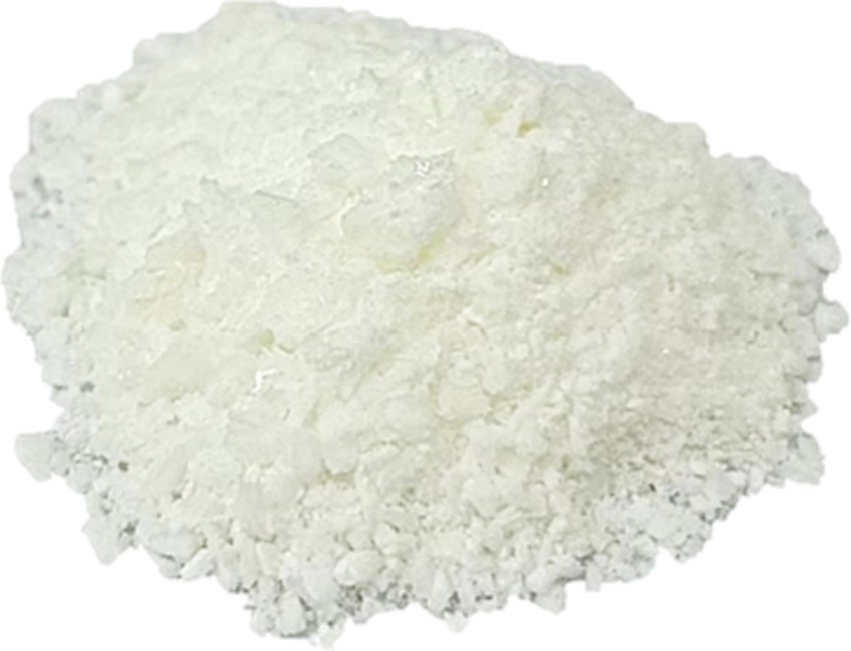
Photograph of CD-GO powder, at 25°C.

After preparation, the CD-GO microcapsules appear pale-yellow granules (Figure 1) with a slight garlic flavor, which is enhanced when dissolved by rehydration. The results of measuring the moisture content (%), bulk density (g/cm3), hygroscopicity (g H2O/100 g), and resting angle θ (°) of CD-GO are shown in Table 1, where the low moisture content prevents lipid oxidation, microbial growth and caking in food products (33). The water content of the CD-GO obtained was low, at 5.86 ± 0.47%. Moisture absorption refers to water adsorption on a solid’s surface. A sample with high moisture absorption is susceptible to decreased mobility, solidification, wetting, and liquefaction and may even promote chemical reactions that reduce the stability of the sample. The hygroscopicity of the prepared CD-GO averaged 5.77 ± 0.53. Refer to a study by Bueno et al. (34) on β-CD encapsulated norfloxacin which classified it as moderately hygroscopic (2–15%). The bulk density and angle of repose of a powder reflect the particle-to-particle space size and the powder-type product’s flowability, respectively, both of which are key parameters affecting storage, transport, handling, packaging, and industrialization (32). The bulk density of the prepared CD-GO was 0.58 ± 0.09 g/mL, and the resting angle θ was 32 ± 4.5°.

**Table 1:** Physical properties of CD-GO.

Moisture content (%)	Bulk density (g/mL)	Hygroscopicity (g H2O/100 g)	Angle of repose θ (°)	EE (%)
5.86 ± 0.47	0.58 ± 0.09	5.77 ± 0.53	32 ± 4.5	94.59

#### 3.1.2 GC–MS analysis

**Figure 2:**
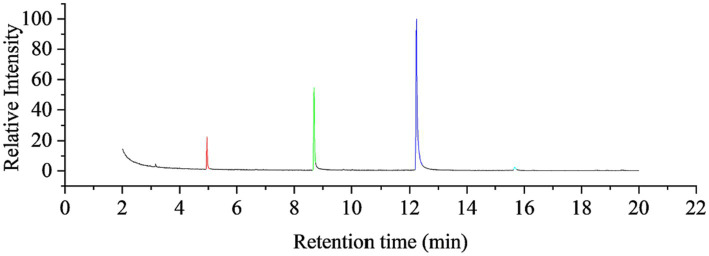
Total ion chromatography chart of GO.

**Figure 3:**
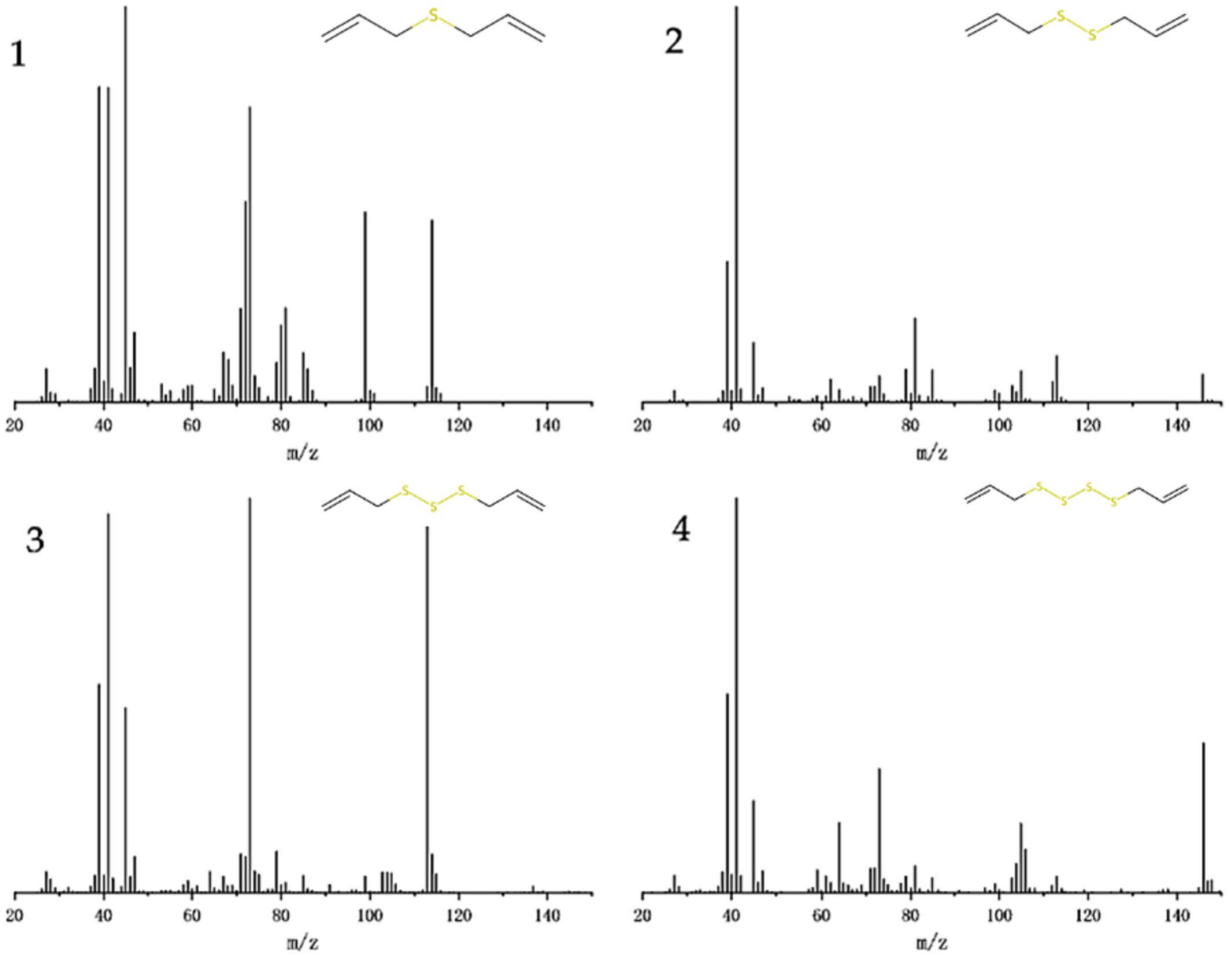
Mass chromatograms of GO, 1 for component 1: diallyl sulfide, 2 for component 2: di-allyl disulfide, 3 for component 3: diallyl trisulfide and 4 for component 4: diallyl tetrasulfide.

GC–MS analyzed the garlic oil, and the total ion chromatography (TIC) results are shown in Figure 2, with characteristic peaks appearing after 4, 8, 12 and 15 min, labeled as fractions 1–4. The mass spectra of the components are shown in Figure 2, which were compared with the Smart SIM database and the specific components with more than 90% similarity are shown in Table 2, Mass chromatograph in Figure 3, diallyl monosulfide, diallyl disulfide, diallyl trisulfide and diallyl tetrasulfide. The standard external method, the standard curve calculated by GC–MS, the equation y = 847,527x-186365. the correlation coefficient R^2 = 0.9995, which correlates well.

**Table 2:** Garlic oil fractions.

Fractions	Retention time (min)	Material	Molecular weight	Relative content (%)
1	4.95	Diallyl sulfide	114	12.54
2	8.69	Diallyl disulfide	146	30.46
3	12.24	Diallyl trisulfide	178	55.75
4	15.67	Diallyl tetrasulfide	210	1.24

#### 3.1.3 Particle size

**Figure 4:**
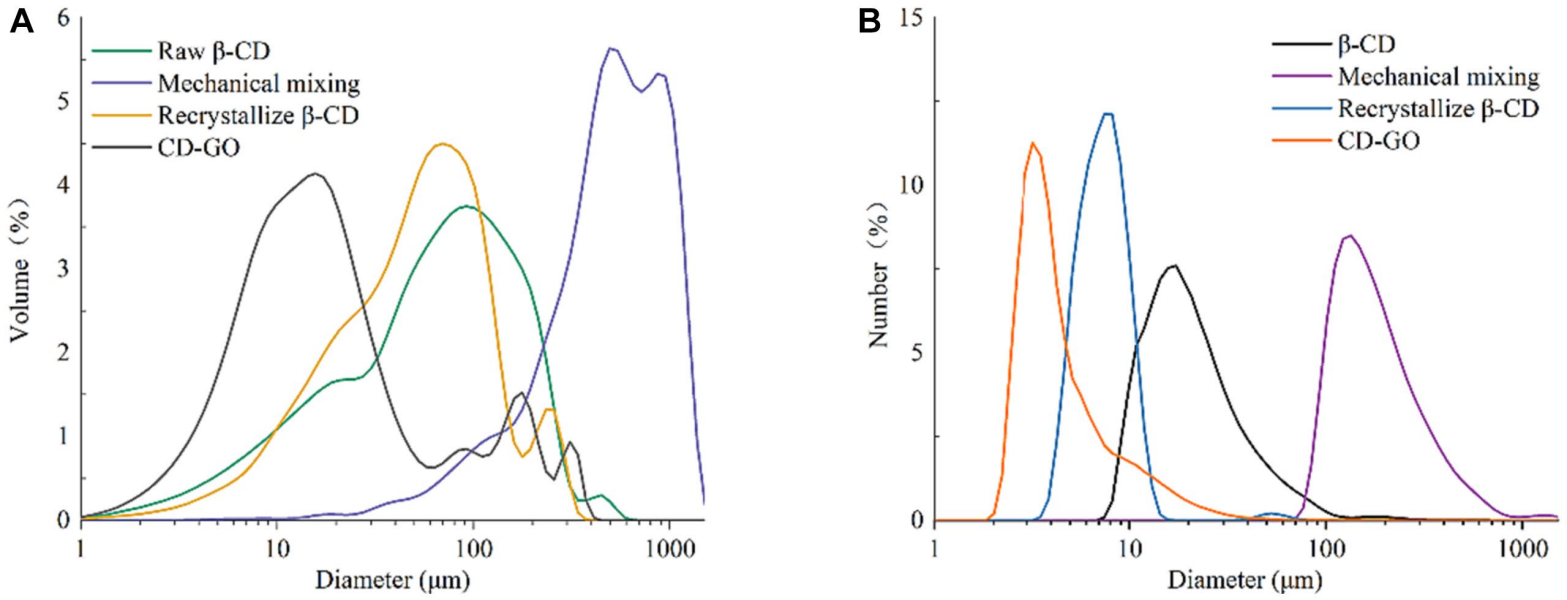
Particle size volume distribution **(A)** and particle size and number distribution **(B)**.

The results of the particle size distribution of β-CD, the physical mixture, recrystallized β-CD, and CD-GO are shown in Figure 4. The volume distribution in Figure 4A shows that the particle size distribution of β-CD is more extensive, which may be due to their interactions. The larger particle size interval of the physical mixture is due to the addition of GO, which adheres to the cyclodextrin surface, further increasing the viscosity and the aggregation between cyclodextrins. Comparing the volume distribution in Figure 4A with the quantity distribution in Figure 4B, the number of small and medium-sized particles is predominant in each group of powders. At the same time, the recrystallized β-CD and CD-GO have a narrower particle size distribution and will have smaller relative particle sizes than the β-CD powders, a result also reflected in a previous result on the particle size of anisaldehyde inclusion compounds (35). The reduction in particle size can be attributed to the process of inclusion preparation, including dissolution, embedding, co-precipitation and filter drying.

#### 3.1.4 Scanning electron microscope

**Figure 5:**
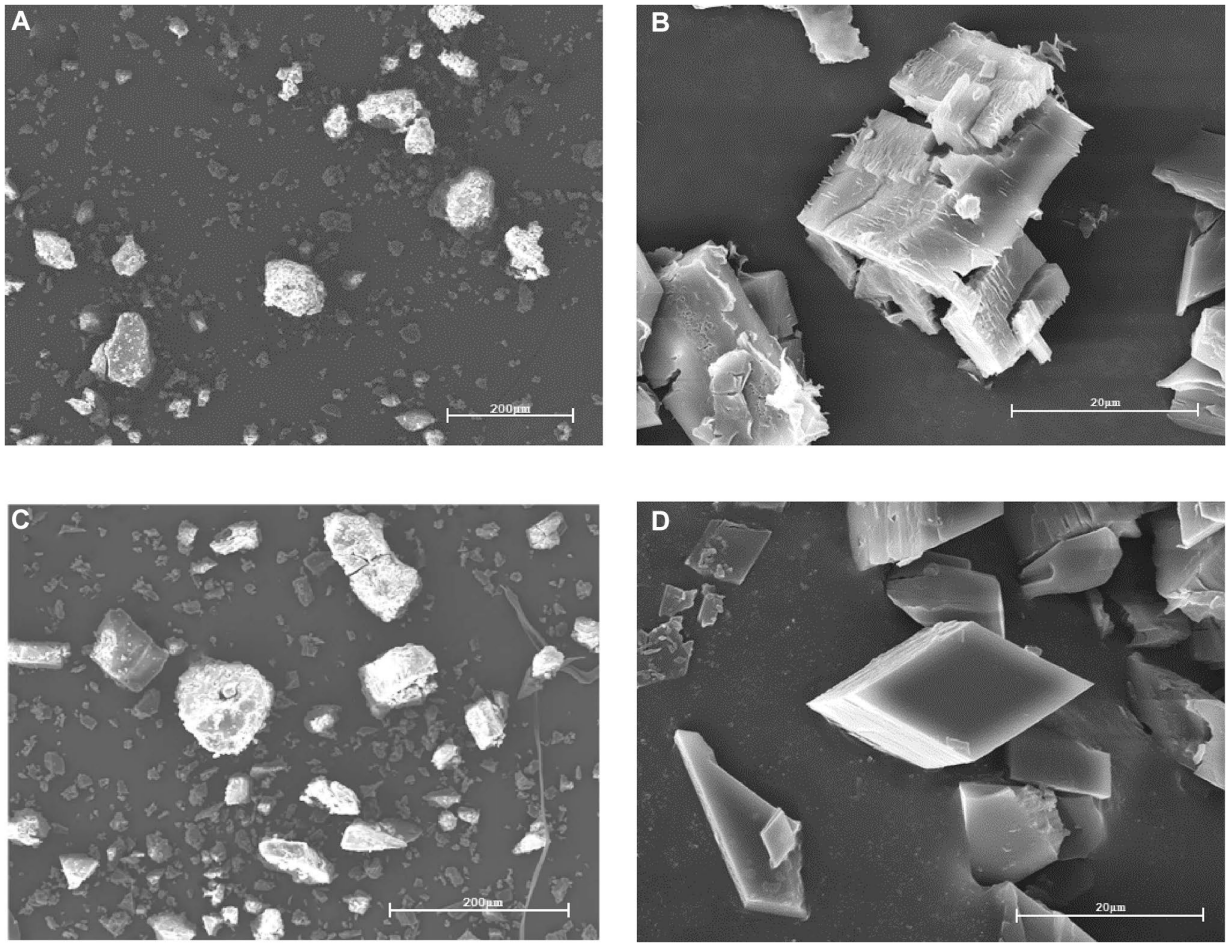
SEM of Raw β-CD **(A)**, recrystallized CD **(B)**, Physical mixture **(C)**, CD-GO **(D)**.

SEM can observe the surface morphology of the material, and the results are shown in Figure 5, Figures 5A–C for the raw β-CD powder, the recrystallized β-CD, and the physical mixture, respectively. However, the CD-GO in Figure 5D shows a significant re-duction in particle size compared to the former, and again the diamond-like lamellar structure is entirely different in shape from the previous three, suggesting the formation of an inclusion complex (36).

#### 3.1.5 FI-TR

**Figure 6:**
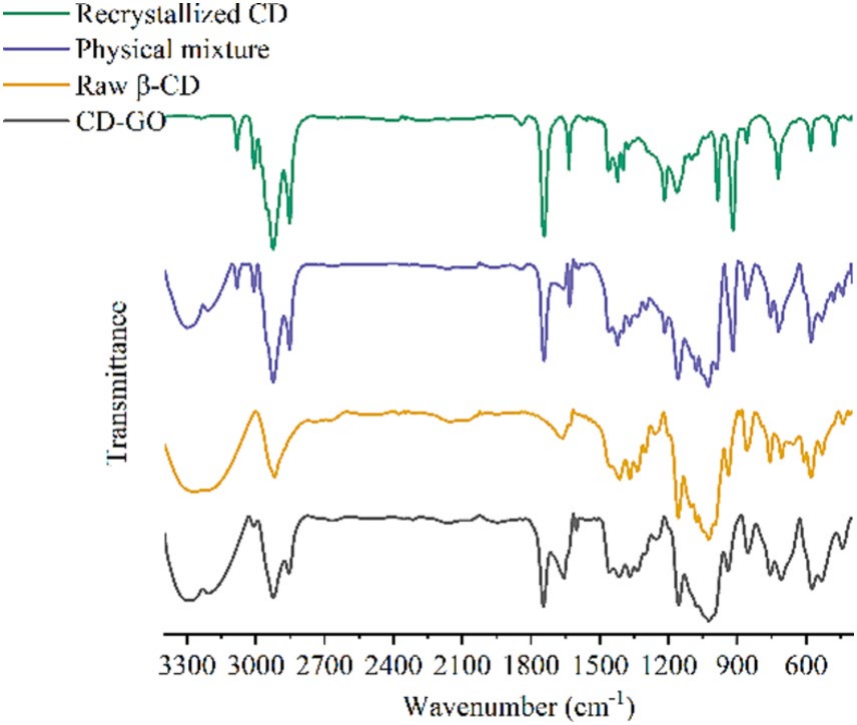
FT-IR spectra of CD-GO, recrystallized CD, Physical mixture.

Infrared spectroscopy is widely used as an analytical tool to identify compounds and the presence or absence of chemical functions and interactions between mixtures. The structure of the infrared absorption profiles detected for GO, physical mixture, CD and CD-GO is shown in Figure 6.

GO spectra, 3,080, 2,920, 2,851 cm-1 for-CH2 stretching vibrations (37) and 1,464, 721 cm-1 for-CH2 bending vibrations, 1,423–1,398 cm-1 and 1,217 cm-1 for-CH2-CH2 = CH-group stretching vibrations, 1,636 cm-1 for C=C stretching vibrations in the propylene group, and 918 cm-1 for strong and narrow peaks for C-S-C stretching vibrations (38).

The CD spectrum shows a characteristic peak for β-CD, with a broad and robust vibration appearing at 3288 cm-1 as a bending of-OH, 2922 cm-1 as a-CH stretching vibration (35), 1,660 cm-1 as a bending of H-O-H, 1024 cm-1 as a symmetric stretching of C-O-C (39) and 1,155 as a bending vibration of-OH (40).

The results of the Physical mixture hybrid spectrograms are like the superimposed GO and CD spectra, where the characteristic peaks of GO are very distinct in the hybrid spectra, indicating a weak or almost non-existent interaction between GO and CD in the physical mixture. However, some of the GO signals are missing or shifted and weakened in the CD-GO spectrum (7, 8), 918 cm-1\u00B0C-S-C stretching vibrations, 1,217 cm-1-and 3,082-CH2 cm-1 stretching vibrations are missing in the CD-GO spectrum, which may be due to GO being encapsulated in β-CD, resulting in a restricted group vibration that weakens its signal This may be because the GO is wrapped inside the β-CD, resulting in a restricted vibration of the group that weakens its signal (41).

#### 3.1.6 X-ray diffraction

**Figure 7:**
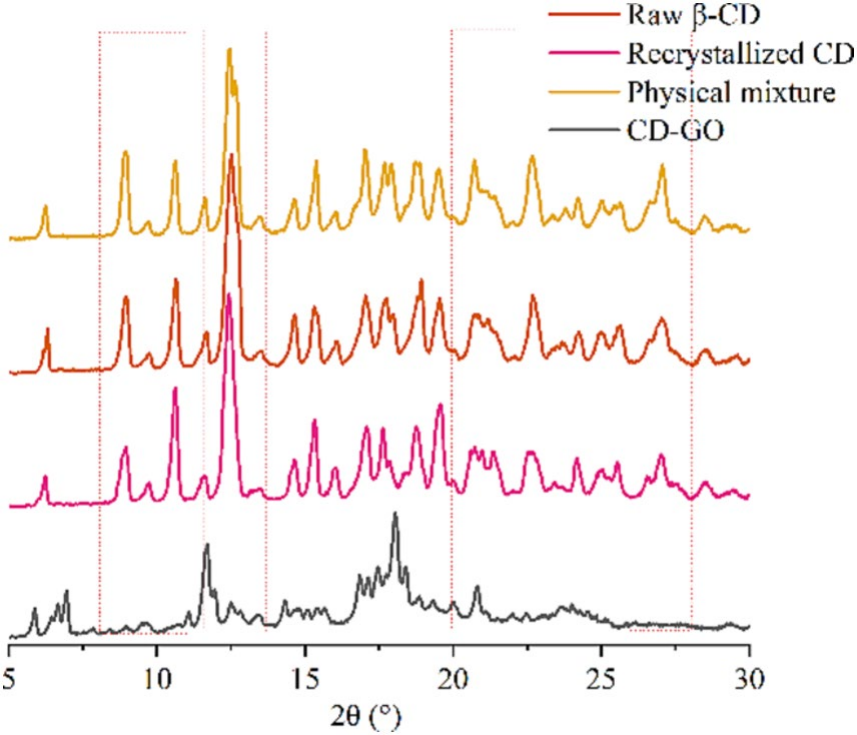
XRD patterns of CD-GO, Recrystallized CD, Physical mixture.

X-ray diffraction is an effective means of detecting β-CD complexation in the powder or micro-crystalline state (42). The crystal structures of β-CD and its inclusions are di-vided into three main categories: channels, cages, and layers. The results of XRD examinations of β-CD, physical mixtures, recrystallized CD and CD-GO are shown in Figure 7. The spectra of β-CD powders show some intense and sharp peaks at diffraction angles of 8.94°, 10.66°, 12.52°, 17.04°, 18.92°, 22.68° and 27.04° at 2θ, and several small peaks at 6.32°, 14.66°, 15.32°, 20.76°, 24.24° and 25.64° at 2θ, confirming that the β-CD has a typical cage-like structure. The diffraction pattern should change significantly if the crystalline shape is affected or altered (43). The diffraction pattern of β-CD recrystallized empty microcapsules showed changes in the intensity of some peaks compared to Physical mixture. No new peaks appeared, indicating that the simple addition of GO Physical mixture did not induce the formation of new compounds or affect the crystal shape of β-CD recrystallized empty microcapsules, like the reported results (35). However, compared to the physically mixed diffraction pattern, some of the peaks present in the physically mixed pattern disappear, maybe because the GO is embedded in the cavity of the β-CD, and the interaction between the subject and the object material leads to different degrees of structural alteration of the β-CD.

#### 3.1.7 Thermogravimetric analysis

**Figure 8:**
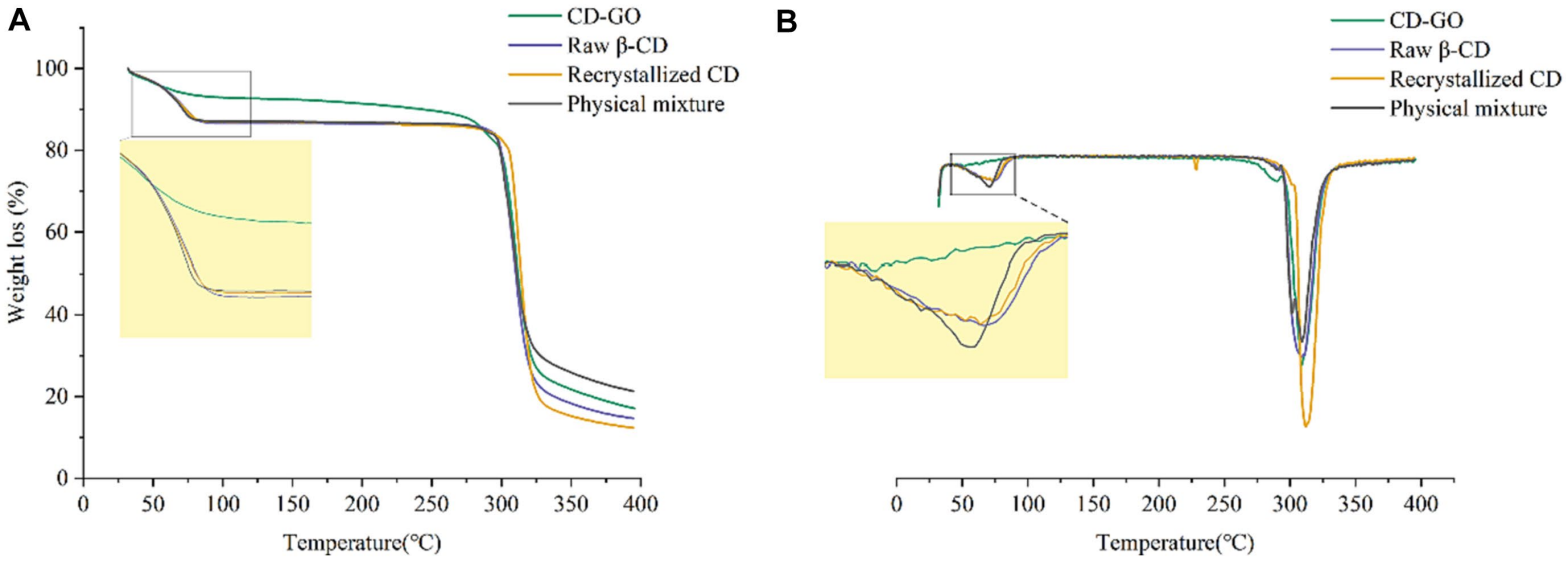
TG diagram **(A)** and DTG **(B)** of CD-GO, Recrystallized CD, Raw β-CD and Physical mixture.

TG measures the mass change of a sample during heating or cooling to determine its thermal decomposition or reaction characteristics. Figure 8A shows TG plots for four different materials, recrystallized CD, β-cyclodextrin powder and physically mixed powder before the first stage at 110°C losing 13.1, 13.4 and 12.9% by weight, respectively, due to evaporation of water from inside the β-cyclodextrin cavity (44). The weight loss of CD-GO in the first stage was around 8.3%. The reason for the slow weight loss of the inclusion complex compared to the other three before the first stage was that since the GO replaced the water molecules inside the cavity of the cyclodextrin and was encapsulated inside the cavity, the temperature in the first stage could evaporate the water on the surface of the cyclodextrin material and the water inside, but not enough to eliminate the GO that replaced the water inside the inclusion complex. The variability of the weight loss graphs in the first stage shows that the GO was successfully encapsulated in-side the cyclodextrin. Taking the first-order derivative of the TG data gives a plot of the variation of DTG in Figure 8B. The DTG plot allows a closer look at the thermal effects of the four different materials. The rate of weight loss is most remarkable in the first phase of the physical blend due to the increased rate of weight loss caused by the rapid evaporation of the volatile components of the garlic oil at elevated temperatures. However, the interaction between the garlic oil components and the inner cavity structure of the β-CD in the inclusion complexes mutually enhances the stability of each (45).

#### 3.1.8 Differential scanning calorimetry

**Figure 9:**
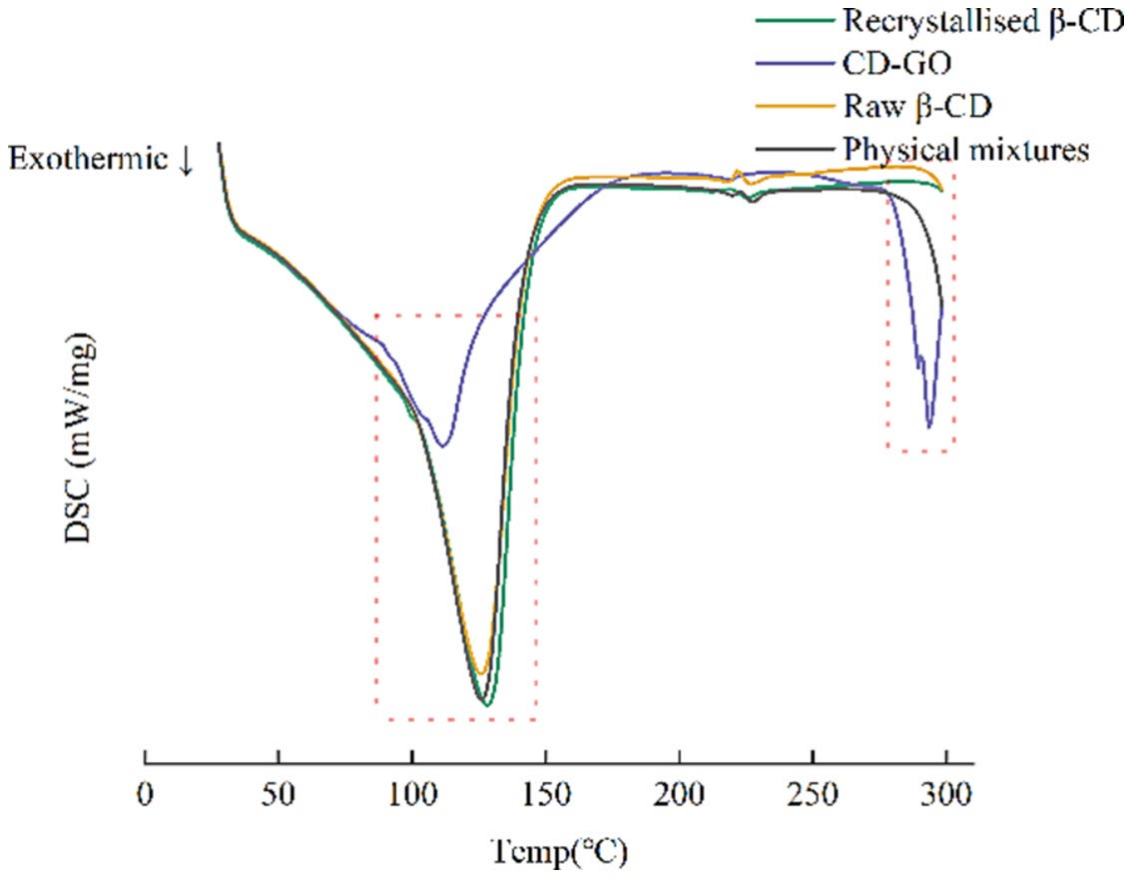
DSC of CD-GO, Recrystallized CD, Raw β-CD and Physical mixture.

DSC allows the thermal analysis of materials to investigate their thermal effects and thermodynamic properties. DSC results are shown in Figure 9. Thus, by combining both techniques, TG and DSC, a more comprehensive understanding of the changes in the properties of the material can be obtained, allowing a more accurate assessment of the outcome of CD-GO inclusion formation. DSC can be used for the identification of inclusion compounds. When guest molecules are embedded in a Raw β-CD cavity, their melting, boiling or sublimation points typically shift to different temperatures or disappear (46). DSC results are shown in Figure 9. CD-GO thermal peak is advanced. This may be due to GO inclusion and CD-GO inside.

#### 3.1.9 Retention and release

**Figure 10:**
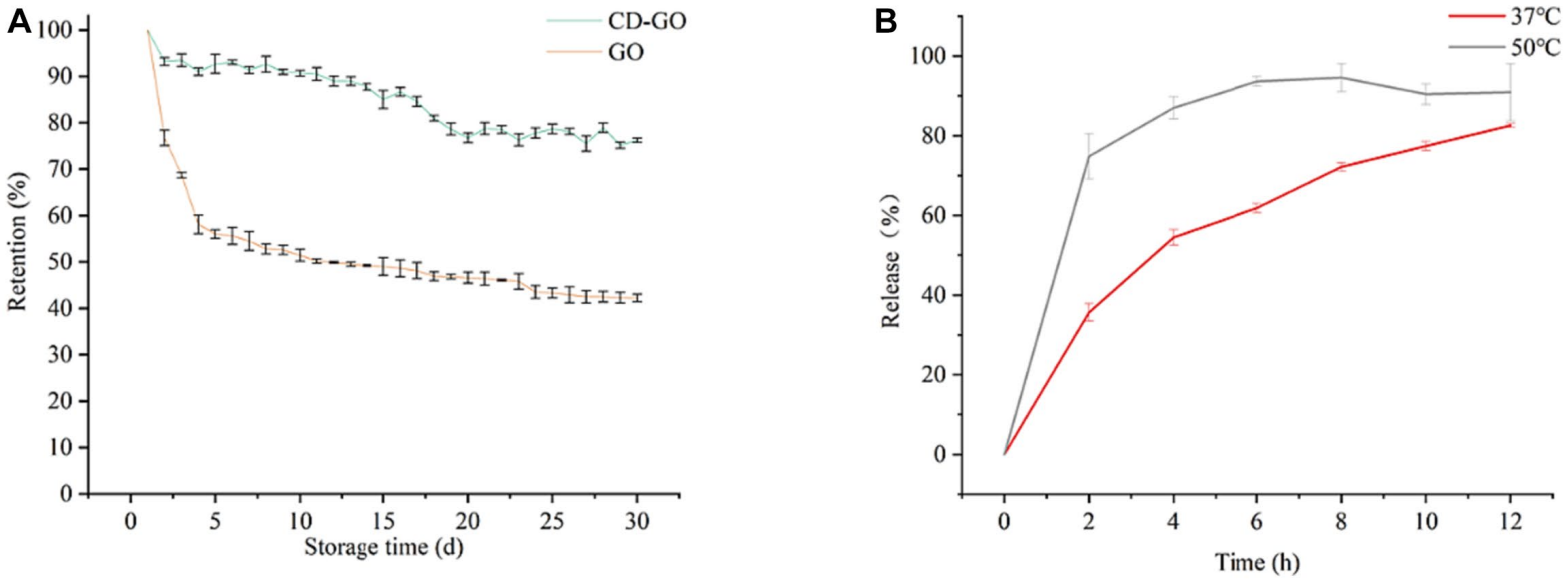
Changes in retention **(A)** of GO and CD-GO with increasing time in open storage at 37°C and 40% humidity; CD-GO release **(B)** *ex vivo* in PBS (pH = 7.4) at 37°C and 50°C. Each value is the mean for three replicates, and vertical bars indicate the standard errors. Each value is the mean for three replicates, and vertical bars indicate the standard errors.

Retention is one of the most important indicators of the performance of a slow-release material and Figure 10A shows the results of the retention of pure GO and CD-GO over time at 37°Ch145% humidity for 30 days. Under the test conditions, a sudden release of GO occurred during the first 4 days and evaporated during the subsequent period, where the mass loss exceeded 50%. After 30 days, only 42.19% of the initial weight remained. In contrast, GO encapsulated in the inclusion complex retained a relatively high percentage during the 30 days of storage, remaining at 76.17% at the end. The release capacity of CD-GO was assayed in PBS, at 37°C and 50°C, and the release pattern is shown in Figure 10B.

At different temperatures, the release pattern of CD-GO was that there was a sudden release at first and then a slow release (37), reaching 54.46% in 4 h at 37°C and 74.82% in 2 h at 50°C. This may be due to the absorption of water by CD-GO, which leads to the change of molecular structure of CD, resulting in the dissolution of part of the inclusion compound, and the release of garlic oil (47). A similar release pattern was seen in the β-CD encapsulation study on anisaldehyde (35).

#### 3.1.10 DPPH clearance rate

**Figure 11:**
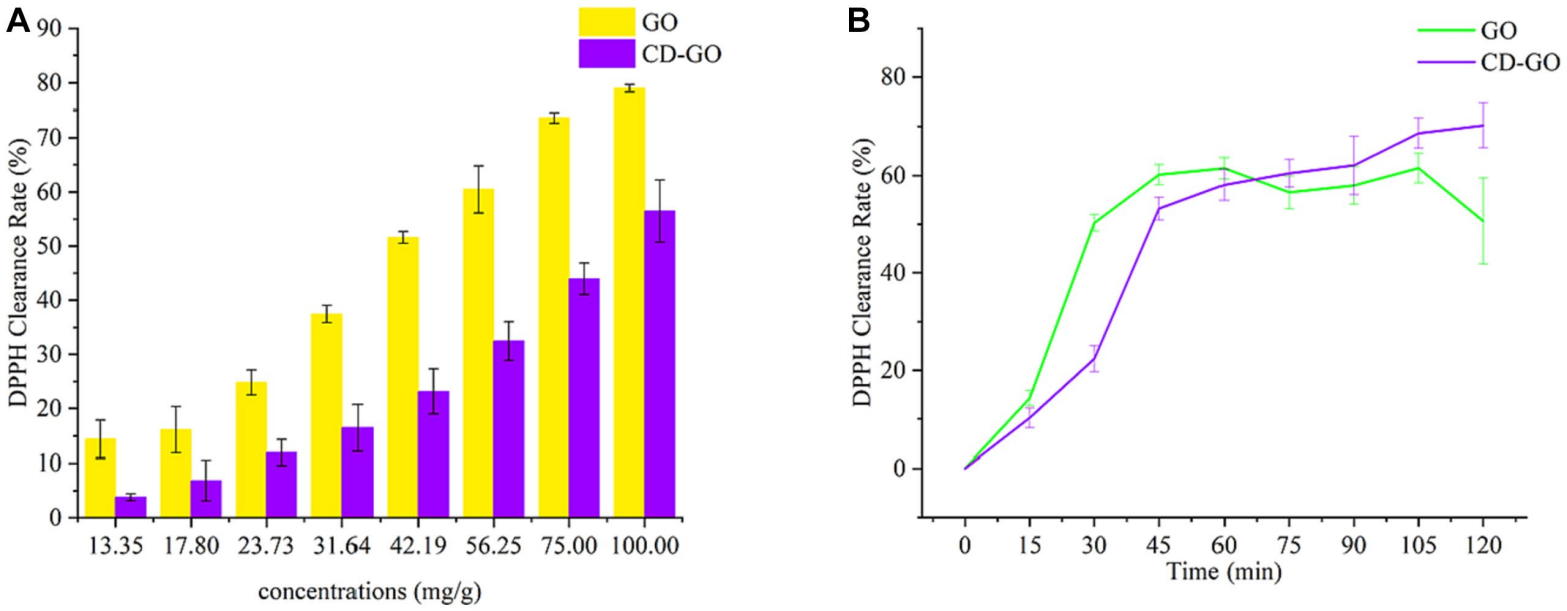
DPPH clearance after 30 min of treatment for samples of different concentrations **(A)** and DPPH clearance at 120 min for samples of 42.19 mg/g **(B)**; Each value is the mean for three replicates, and vertical bars indicate the standard errors.

The results of DPPH radical scavenging by CD-GO are shown in Figure 11. Figure 11A shows the DPPH scavenging rate after 30 min of treatment for different concentrations of samples, with increasing antioxidant activity as the concentration of samples increased. At a 75 mg/g concentration, the DPPH scavenging rate of GO reached 73.62%, while that of CD-GO was at 43.96%. Figure 11B shows the results of the change in DPPH clearance over 120 min for the 42.19 mg/g sample, with the cumulative DPPH clearance increasing for both samples as time increased. For the first 75 min, GO was higher than CD-GO regarding DPPH clearance and clearance rate. However, as the time exceeded 75 min, the DPPH clearance in the GO group stopped increasing while CD-GO continued to increase until it reached 70.22% DPPH clearance at 120 min, which was 17.57% higher than GO at 1.32 times the DPPH clearance rate of GO. Existing studies have shown that β-CD alone is not reductive (48), so the reason why CD-GO is more reductive than GO may be that the reducing sulfide component of GO is encapsulated by CD, increasing its solubility and stability, and preventing its volatilization or deactivation. CD-GO, there-fore, exhibits a sustained antioxidant capacity compared to GO. A similar phenomenon was found in encapsulated pairs of anisaldehyde cyclodextrins (35).

### 3.2 Meatloaf application

#### 3.2.1 Texture profile analysis

**Figure 12:**
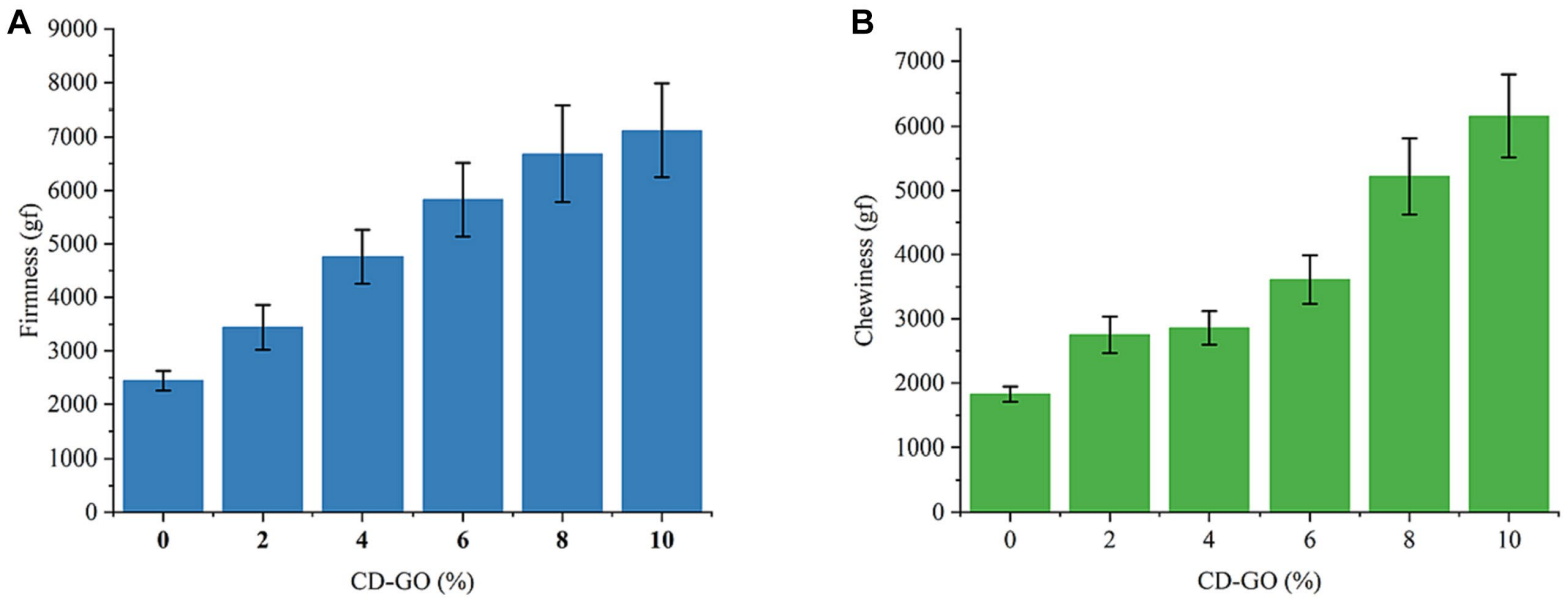
Effect of CD-GO on firmness **(A)** and Chewiness **(B)** of plant-based meat; Each value is the mean for three replicates, and vertical bars indicate the standard errors.

Firmness is the most direct indicator of mouthfeel, and in texture profiling, it directly affects chewiness, chewiness, Springiness, and Gumminess (49). The results of the effect of CD-GO on the firmness of plant-based flesh are shown in Figure 12A. The results showed that the firmness of plant-based meat increased with CD-GO addition. Due to the strong adhesive force of dextrin, the plant-based tissue proteins were made to form a dense fibrous mesh structure with each other leading to an increase in the firmness of plant-based meat patties. Chewiness is defined as adhesion multiplied by elasticity. It can be interpreted as the energy required to chew solid food (50). Figure 12B shows the effect of CD-GO on plant meat Chewiness. The results showed that CD-GO increased the chewiness of meatloaf.

#### 3.2.2 Meatloaf structure

**Figure 13:**
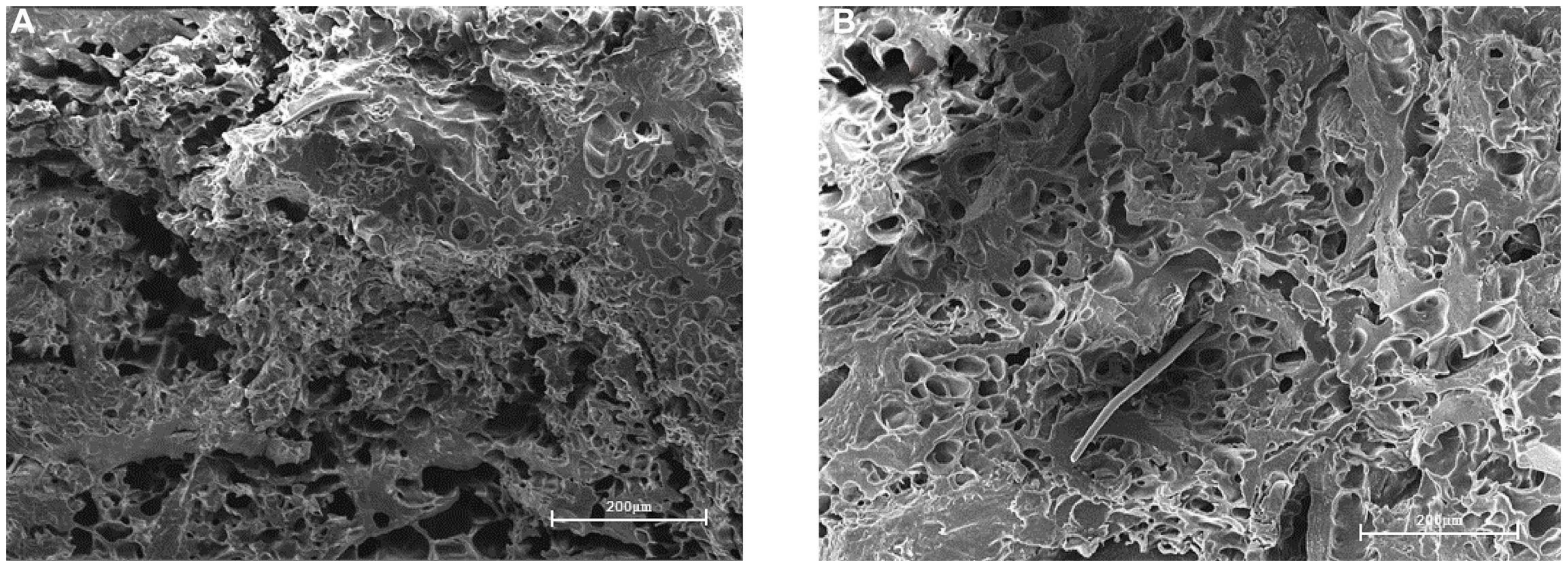
SEM of CD-GO-free plant-based meat **(A)**, Plant-based meat with 10% CD-GO **(B)**.

Figure 13 shows a plant-based meatloaf under a scanning electron microscope magnified 300 times. Figure 13A shows the meat without added CD-GO, where more gaps are visible and unevenly distributed. Figure 13B is meat with 10% CD-GO added, with a lower number of crevices and a uniform distribution of holes in the cut surface, visible as the formation of a reticulated structure, consistent with the TPA’s speculation that the addition of CD-GO increases the hardness and chewiness of the meat as a result of the formation of a reticulated structure by the dextrin. Adding CD-GO contributes to a more stable and delicate structure of plant-based meat.

#### 3.2.3 Sensory evaluation

**Figure 14:**
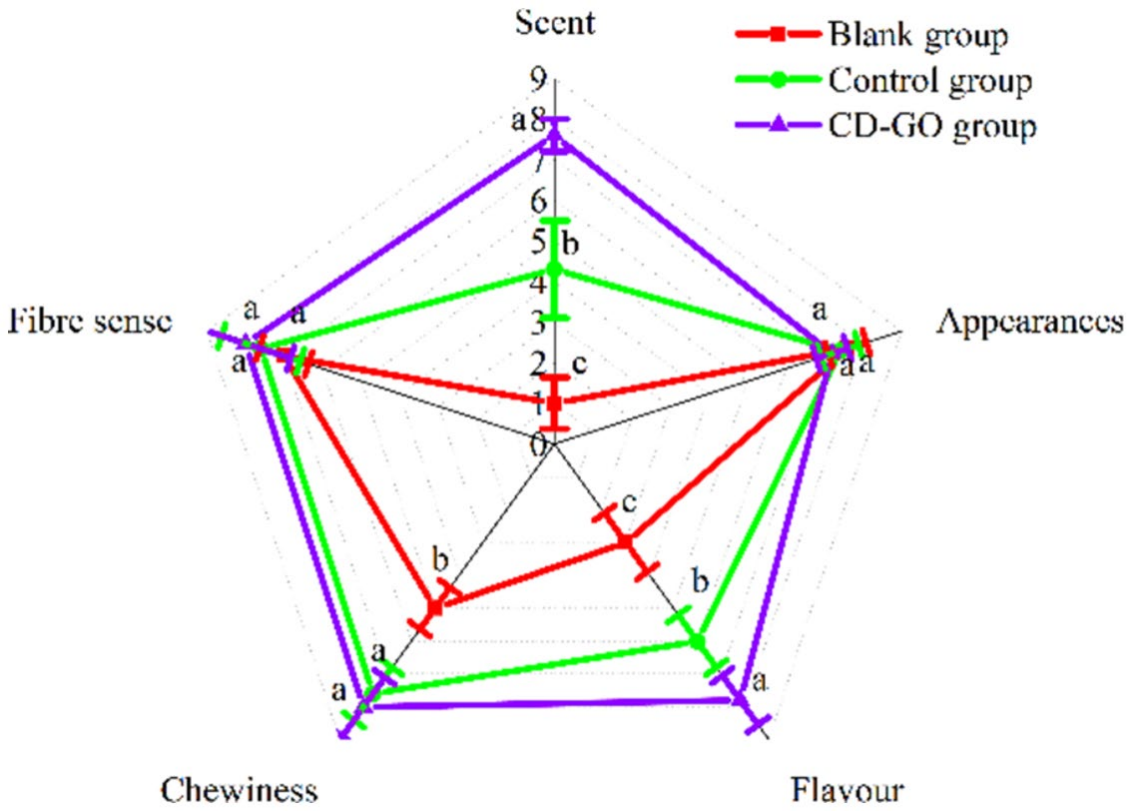
Blank group was pure plant patties, control group was plant patties supplemented with 10% Physical mixtures, and CD-GO group was plant patties supplemented with 10% garlic oil cyclodextrin microcapsules. The nine-point hedonic evaluation scale was used as follows: 9, like very much to 1, dis4.3like very much. For each sample, the means designated by different letters are significantly different (*p* < 0.05).

Essential oils robust and pungent odor and taste can affect people’s interest in food and its organoleptic qualities (51). The sensory evaluation results are shown in Figure 14 and demonstrate that with or without the addition of CD-GO, it had little effect on the overall appearance and fiber feel of the plant-based meat, scoring within 7–8 like moderately and like a lot (*p* > 0.05). In terms of odor, the CD-GO group scored the highest at 7.6, followed by the control group with 4.3, and the worst was the blank group with 1. because the garlic oil in the control group was mixed directly into the plant-based meat. The encapsulation of the microcapsules did not mask the odor, and the overpowering odor led to a decrease in the score. The same is reflected in the results of the flavor score item. Finally, the chewiness score showed that both the CD-GO and control groups were close to each other with scores between 7 and 8, indicating that garlic oil at this concentration does not affect the chewiness of the patties; it is the cyclodextrin that affects the chewiness. Adding 10%, CD-GO can effectively improve the flavor and texture of plant-based meat and enhance the value of plant-based meat patties.

## 4 Discussion

Demand for plant-based meat will likely increase as more people become aware of the environmental and health impacts of meat consumption (52). Additionally, population growth and inefficiencies in meat production will challenge traditional meat production. Consequently, plant-based meat represents a potentially sustainable alternative to meat consumption. The growth of the plant-based meat industry will be shaped by a range of factors. Garlic oil is an excellent flavoring agent. However, garlic oil has the disadvantages of low water solubility and poor stability (53). To solve these problems, β-CD was chosen to encapsulate garlic oil to improve the water solubility and stability of garlic oil for better application in plant-based meat.

At present, the popular preparation methods of cyclodextrin inclusion compounds include saturated aqueous solution method, milling method, freeze drying method, spray drying method (54). Cyclodextrin inclusion complexes prepared by the milling method could not be produced on a large scale. The preparation of cyclodextrin inclusion complexes by lyophilization and spray-drying methods requires expensive and large-scale equipment. In the present study, garlic oil cyclodextrin inclusion complexes were prepared and characterized by saturated co-precipitation synergistic ultrasonic microwave method. The results of the characterization analysis showed that CD-GO was a light-yellow powder with a size of about 10 μm, and its rhombic particles were visible under SEM, which clearly distinguished it from the unencapsulated cyclodextrin (36). Raw β-CD has a typical cage-like structure, and after XRD analysis, the CD-GO pattern is obviously different from that of raw β-CD, which may be caused by the presence of guest substances that affect the crystalline structure of cyclodextrins (35). The TG results show that the initial weight loss of CD-GO is slowed down again, demonstrating that the inclusion can effectively protect GO in the heat flow, a result like that of an *Illicium verum* essential oil study (Wu et al., 2022). At the same time, CD-GO also has a good ability to retain GO, which can make GO release in water stable. A similar release pattern was seen in the β-CD encapsulation study on anisaldehyde (35). We found that encapsulation of GO in CD-GO still retained the excellent antioxidant ability of GO, while CD-GO exhibited more powerful scavenging ability than GO in DPPH scavenging experiments (35). Finally, the results showed that the encapsulated garlic oil still had antioxidant ability and slow-release properties. The final addition to Plant-based meat gives them a delicious flavor and adds texture and mouthfeel.

## 5 Conclusion

A CD-GO inclusion complex was prepared in this thesis by successfully encapsulating GO inside β-CD using a saturation co-precipitation method. The CD-GO inclusion complex was found to have a moisture content of 5.86%, a bulk density of 0.58 g/mL, an inclination angle of 32°, and a hygroscopicity of 5.77 H2O/100 g, with 94.59% encapsulation. A total feed of 11% CD and GO products yielded up to 91%, with a total volume of 1 L. Analysis of TG, DSC, FI-RT, and XRD results indicated a successful preparation of the CD-GO inclusion complex. The study examined the retention rate and revealed that the inclusion compound increased it by 33.98% compared to pure GO. The releasability assay demonstrated that the inclusion complex was released effectively in PBS at 37°C pH = 7.4. According to the free radical scavenging assay, the inclusions exhibited similar antioxidant ability to free GO, while 42.19 mg/g inclusions displayed enhanced and long-lasting antioxidant potency compared to free GO at the same concentration. Lastly, the findings of the meatloaf application study demonstrate that using CD-GO resulted in increased cohesion, firmness, and enhancement of flavor and texture in the meatloaf, leading to im-proved organoleptic evaluation.

## Data availability statement

The original contributions presented in the study are included in the article/supplementary material, further inquiries can be directed to the corresponding author.

## Author contributions

SL: Conceptualization, Data curation, Methodology, Software, Validation, Writing – original draft, Writing – review & editing. JC: Formal analysis, Software, Validation, Writing – review & editing, Writing – original draft. YL: Investigation, Software, Writing – review & editing. HQ: Software, Validation, Writing – review & editing. WG: Investigation, Visualization, Writing – review & editing. KC: Writing – review & editing, Visualization. BZ: Writing – review & editing, Formal analysis. RL: Writing – review & editing, Software. WH: Funding acquisition, Project administration, Resources, Supervision, Writing – review & editing.
